# A formative evaluation of the implementation of an upper limb stroke rehabilitation intervention in clinical practice: a qualitative interview study

**DOI:** 10.1186/s13012-014-0090-3

**Published:** 2014-08-12

**Authors:** Louise A Connell, Naoimh E McMahon, Jocelyn E Harris, Caroline L Watkins, Janice J Eng

**Affiliations:** Clinical Practice Research Unit, School of Health, University of Central Lancashire, Preston, PR1 2HE UK; School of Rehabilitation Science, McMaster University, 1400 Main Street West, Hamilton, Ontario L8S 1C7 Canada; Department of Physical Therapy, University of British Columbia, 212-2177 Wesbrook Mall, Vancouver, V6T 1Z3 British Columbia Canada

## Abstract

**Background:**

The Graded Repetitive Arm Supplementary Program (GRASP) is a hand and arm exercise programme designed to increase the intensity of exercise achieved in inpatient stroke rehabilitation. GRASP was shown to be effective in a randomised controlled trial in 2009 and has since experienced unusually rapid uptake into clinical practice. The aim of this study was to conduct a formative evaluation of the implementation of GRASP to inform the development and implementation of a similar intervention in the United Kingdom.

**Methods:**

Semi-structured interviews were conducted with therapists who were involved in implementing GRASP at their work site, or who had experience of using GRASP. Normalisation Process Theory (NPT), a sociological theory used to explore the processes of embedding innovations in practice, was used to develop an interview guide. Intervention components outlined within the GRASP Guideline Manual were used to develop prompts to explore how therapists use GRASP in practice. Interview transcripts were analysed using a coding frame based on implementation theory.

**Results:**

Twenty interviews were conducted across eight sites in British Columbia Canada. Therapists identified informal networks and the free online availability of GRASP as key factors in finding out about the intervention. All therapists reported positive opinions about the value of GRASP. At all sites, therapists identified individuals who advocated for the use of GRASP, and in six of the eight sites this was the practice leader or senior therapist. Rehabilitation assistants were identified as instrumental in delivering GRASP in almost all sites as they were responsible for organising the GRASP equipment and assisting patients using GRASP. Almost all intervention components were found to be adapted to some degree when used in clinical practice; coverage was wider, the content adapted, and the dose, when monitored, was less.

**Conclusions:**

Although GRASP has translated into clinical practice, it is not always used in the way in which it was shown to be effective. This formative evaluation has informed the development of a novel intervention which aims to bridge this evidence-practice gap in upper limb rehabilitation after stroke.

**Electronic supplementary material:**

The online version of this article (doi:10.1186/s13012-014-0090-3) contains supplementary material, which is available to authorized users.

## Background

In recent years, there has been a dramatic increase in stroke rehabilitation research and the evidence-base has grown exponentially. High-intensity, repetitive, task-oriented training demonstrates the best evidence for improving motor recovery after stroke [1-4]. However, it is known that stroke rehabilitation, in its present form, is not achieving the required intensity to maximise recovery after stroke [5,6].

The Graded Repetitive Arm Supplementary Program (GRASP) is one method of increasing intensity of exercise during inpatient rehabilitation. In 2009, GRASP was shown to be significantly more effective in promoting functional recovery of the upper limb after stroke compared to usual care [7]. GRASP is a self-directed hand and arm exercise programme that is taught and monitored by a therapist, but carried out by the patient with the support of their family/carer where possible. The program is not meant to replace existing therapy services, rather to augment current therapy, adding opportunities for more practice. Despite only one randomised controlled trial (RCT) having demonstrated the efficacy of GRASP, a recommendation to ‘provide a graded repetitive arm supplementary program for patients to increase activity on ward and at home’ was included in the 2010 update of the Canadian Best Practice Recommendations for Stroke Rehabilitation [8]. Anecdotally GRASP is reported to be used in over 30 centres in Canada [9] and from a sample of 274 therapists in the United Kingdom (UK), over 40% had heard of GRASP and almost one-quarter had experience of using GRASP in practice [10].

As the long-term objective of this work is to develop a feasible and structured upper limb exercise programme for use in UK stroke rehabilitation units, it is of value to learn from the implementation of GRASP. Stetler *et al.* [11] have highlighted the role of formative evaluation in implementation research, defining it as ‘a rigorous assessment process designed to identify potential and actual influences on the progress and effectiveness of implementation efforts.’ There is growing interest in being able to systematically explore and explain the implementation of evidence-based interventions and this has resulted in the development a number of theoretical frameworks. It has been suggested that the use of such frameworks will help advance implementation research by providing consistency in definitions and terminologies across contexts [12], and by providing systematic explanations of phenomena and constructs that influence implementation [13]. There is also increasing emphasis being placed on evaluating implementation fidelity [14], as each time implementation of an intervention is attempted, there is an opportunity to learn about conditions that result in better or worse fidelity, in order to assist refinement [15].

The aim of this study was to conduct a formative evaluation of the implementation of the Graded Repetitive Arm Supplementary Program (GRASP) in Vancouver, British Columbia (BC), Canada to inform the development and implementation of a similar intervention in the UK.

The objectives of this study were to use semi-structured interviews to:

1. Explore how therapists found out about GRASP.
2. Explore the processes that therapists’ report were involved in implementing GRASP in practice.
3. Explore therapists’ experiences of using GRASP in clinical practice and how this adheres to intervention components outlined within the GRASP Guideline Manual.
4. Use a taxonomy of factors influencing implementation to explain the research findings.

## Methods

### Research team and reflexivity

The first author (LAC) and second author (NEM) conducted the interviews. Both are female-chartered physiotherapists with previous experience of qualitative data collection. Both hold full-time research positions at a UK Higher Education Institution working on a National Institute for Health Research (NIHR) funded project to develop a clinically feasible structured upper limb exercise programme for use in National Health Service (NHS) stroke rehabilitation units. The researchers were not known to the participants prior to the study. Participants were informed in the first recruitment email that two researchers from the UK were exploring how GRASP has been implemented in practice. The third (JH) and last (JE) author developed GRASP and conducted the randomised trial confirming its effectiveness [7]. The fourth (CW) author is a health services researcher with experience in implementation science.

### Study design

A cross-sectional study design was used with data collected via semi-structured interviews.

### Theoretical framework

The approach used in this study was directed content analysis, a qualitative approach that is guided by a structured process underpinned by theory [16]. Three frameworks from implementation science were used to address the study objectives and are detailed below.

### Normalisation Process Theory (NPT)

Normalisation Process Theory (NPT) is a sociological theory that can be used to understand the implementation, embedding, and integration of innovation in healthcare settings [13]. NPT is made up of four constructs each of which has four components: coherence is the first construct, and describes the sense-making processes that people go through when introduced to a new innovation; cognitive participation describes the process of committing to implementing the innovation; collective action describes how the work to implement the intervention gets done; and reflexive monitoring describes the evaluation work that takes place. The emphasis of these components is on the dynamic and interactive processes that take place when attempting to embed a new innovation or practice. A recent systematic review found that in most cases NPT has been used as an organising framework for analyses and reporting of findings in health research [17]. It has also been used to inform study/intervention design, to generate research questions for fieldwork, and to create tools for investigating and supporting implementation [17]. In this study, NPT was used in developing the interview guide and in data analysis to explore the processes involved in identifying, integrating, and embedding GRASP in practice.

### Conceptual Framework for Implementation Fidelity (CFIF)

Carroll *et al.* developed the Conceptual Framework for Implementation Fidelity (CFIF) to guide the measurement of implementation fidelity [18]. Within this framework, the elements of implementation fidelity are: coverage (who should be receiving the intervention); content (the intervention itself); and dose (duration and frequency of the intervention). The degree to which these elements are delivered can be influenced by moderating factors, *e.g.*, intervention complexity, facilitation strategies, and participant responsiveness. The CFIF has previously been used empirically to evaluate fidelity [14,19]. In this study, the CFIF was used to analyse interview transcripts to explore the coverage, content, and dose when GRASP is used in clinical practice, and how this adheres to intervention components outlined within the GRASP Guideline Manual.

### Consolidated Framework for Implementation Research (CFIR)

The Consolidated Framework for Implementation Research (CFIR) has been developed by Damschroder *et al.* and is a pragmatic taxonomy of the factors that influence implementation [12]. CFIR has five domains (characteristics of the intervention, inner setting, outer setting, characteristics of individuals, and processes), each of which contain a number of constructs. The framework can be used to guide assessments of implementation, evaluate implementation progress, and explain findings in research studies [12]. In this study, CFIR was used in data analysis to identify emerging factors that influenced implementation and use of GRASP, and to propose potential explanations for the research findings.

### Participant selection

A purposive sample of physical therapists, occupational therapists and rehabilitation assistants: who were currently using GRASP, or had previous experience of using GRASP, or who were involved in the implementation of GRASP at their work setting were recruited to take part in this study. Therapists and work settings that were not using GRASP or did not have experience of implementing GRASP in practice were not eligible for inclusion. Potential participants were identified through existing contacts with the research team (*e.g.*, through sites involved in the GRASP RCT), through the public registries for BC Occupational Therapists and Physical Therapists and through a database of therapists that had agreed to be contacted about future research relating to the program through the GRASP website. These potential participants were sent an email by the fifth author (JE) outlining the details of the study and inviting them to take part in an interview of maximum one hour in length. A snowball sampling technique was used in which these participants identified colleagues from their own work place, or from other sites in the region, who would be suitable to take part.

### Setting

The interviews were conducted by the first (LAC) and second author (NEM). Interviews took place at the work site of participants at a time deemed suitable by them. In instances where it was not possible to conduct the interviews face-to-face, the interviews were carried out over the telephone.

### Data collection

The data collection tool used in this study was an interview guide (see Additional file 1: Interview Guide). NPT was used to devise questions and prompts about the processes of implementing and embedding GRASP in practice. Following introductory questions, participants were asked how they found out about GRASP and to describe in their own words how they use GRASP in practice. The GRASP Guideline Manual (http://neurorehab.med.ubc.ca/grasp/) was used to identify components of GRASP against which fidelity could be evaluated, and these were included as prompts within the interview guide. For example, in the GRASP Guideline Manual pg.8 line 9 the instruction given is ‘Show patient and family how to do each exercise.’ A prompt relating to the family and carer involvement component of GRASP was developed for inclusion in the interview guide. The interview guide was reviewed and piloted with researchers (n = 2) with previous experience of using implementation frameworks for semi-structured interviews, and with therapists (n = 3). The interviews lasted a maximum of one hour. They were audio-recorded and field notes made. All participants provided written informed consent and received a $25 Canadian honorarium to compensate them for their time. Interviews were conducted until no new implementation issues were being reported and data saturation was deemed to have been reached.

### Data analysis

Interview transcripts were transcribed verbatim and imported into NVivo 10 for analysis. Transcripts were first read for understanding to describe each case and to establish an initial coding frame. The coding frame was also informed by prior research that explored upper limb exercise prescription by UK therapists, and uptake of GRASP in the UK, as it was hypothesised that similar experiences would arise for both population groups [10,20]. Transcripts were then re-read by the first and second authors and separately coded.

The coding frame evolved as analysis progressed (see Additional file 2: Coding Frame). This was facilitated by regular team meetings to discuss and agree on emerging themes and resolve discrepancies in coding. NPT constructs were used to code text relating to the processes of implementing GRASP in clinical practice. The CFIF was used to code text relating to how GRASP is used in practice. These codes were then used to evaluate adherence to the intervention components identified *a priori* from the GRASP Guideline Manual. The CFIR was used to code emerging factors that influenced both use and implementation of GRASP. Therapists in the research team provided feedback throughout the process, which helped to ensure that findings were credible.

### Ethical approval

This study was approved by the University of British Columbia Behavioural Research Ethics Board (BREB), study number H13-00249.

### Findings

In total 42 potential participants across 12 sites were invited to take part. Of these, 23 replied to the email invite, and 20 therapists from eight different sites agreed to take part in an interview (two were not using GRASP and one replied after data collection had ceased). Non-participants did not reply to the email invite. The reasons for non-participation are therefore unknown and it is not possible to determine whether or not non-responders were implementing GRASP. Participant characteristics are shown in Table 1 along with their anonymised identification codes. For details on individual recruitment across sites see Additional file 3: Recruitment of participants. Therapists from eight of the twelve contacted sites participated. Seven of these sites were regional hospitals and one site was a rehabilitation centre. Two of the eight sites were situated in the Greater Vancouver area.

**Table 1:** **Participant characteristics**

**Site**	**Professional title**	**Years of experience**	**Level of education**	**Introduction to GRASP**	**Experience with GRASP**
A	Occupational therapist (#OT1)	9	BSc	RCT	Involved in RCT, currently using GRASP in community setting
	Occupational therapist (#OT2)	3	MSc	University	Involved in implementing GRASP in acute care setting, currently using GRASP in inpatient rehabilitation
	OT practice leader (#OT3)	30	BSc	Colleagues/work in-service	Involved in implementing GRASP at site A
	Occupational therapist (#OT4)	25	BSc	Colleagues	Previous experience of using GRASP in inpatient rehabilitation, not using GRASP in current role
	PT practice leader (#PT1)	36	BSc	Research Team at GF Strong	Involved in implementing GRASP at site A
B	PT practice leader (#PT2)	11	BSc	Physiotherapy Forum	Involved in implementing GRASP at site B, currently using GRASP in groups in inpatient rehabilitation
C	Occupational therapist (#OT5)	6	BSc	Colleagues/work in-service	Has experience of using GRASP in inpatient rehabilitation
	OT practice leader (#OT6)	22	MSc	Colleagues/work in-service	Involved in implementing GRASP at site C
D	Physiotherapist (#PT3)	5	BSc	Colleagues/work in-service	Has experience of using GRASP in acute, inpatient rehabilitation and outpatient settings
E	Occupational therapist (#OT7)	12	BSc	Colleagues/work in-service	Using GRASP in acute care and inpatient rehabilitation
	Rehabilitation assistant (#RA1)	6	Cert	Colleagues/work in-service	Using GRASP in inpatient rehabilitation, has experience of using GRASP in outpatients
	Occupational therapist (#OT8)	19	BSc	Colleagues/work in-service	Using GRASP in inpatient rehabilitation
	Rehabilitation assistant (#RA2)	8	Cert	Colleagues/work in-service	Using GRASP in inpatient rehabilitation
	Occupational therapist (#OT9)	8	MSc	Research Team at GF Strong	Using GRASP in outpatients
F	Occupational therapist (#OT10)	>5	BSc	Colleagues/work in-service	Using GRASP in outpatients
	Physiotherapist (#PT4)	3	MSc	University	Using GRASP in acute care
	Physiotherapist (#PT5)	4	MSc	Colleagues/work in-service	Using GRASP in acute care
G	Occupational therapist (#OT11)	>5	BSc	Own research	Using GRASP in community setting
	OT practice leader (#OT12)	37	BSc	Own research	Involved in implementing GRASP at site G
H	Occupational therapist (#OT13)	15	BSc	RCT	Involved in RCT and in implementing GRASP at site H

### How therapists found out about GRASP

The way in which each therapist found out about GRASP is shown in Table 1. Two therapists had acted as site co-ordinators for the RCT; 11 therapists found out about GRASP through colleagues or work in-services; one therapist learned about GRASP at a national physiotherapy forum; two therapists learned about GRASP as students in university; two therapists found out about GRASP through their own research; and two therapists found out about GRASP through the research team at GF Strong.

### Processes involved in implementing GRASP in practice

#### Coherence

First impressions of GRASP were predominantly positive with almost all therapists expressing that they felt GRASP was well supported by the evidence base, was well presented and that it was something that would help them in their role:

‘I remember being impressed just by the research findings, so I remember that was highlighted that it did show the extra practice on top of therapy sessions did have significant results so I think I was just am felt happy about the research findings that they had from the study.’ #OT2

‘I thought it was great, I like that there was big pictures, the writing is big, it’s very well laid out, very easy to give out as a home programme once you introduce somebody to it and then to give to them to do on their own.’ #PT3

However, all therapists interviewed also expressed some concerns about the quality of exercises that patients would be able to complete outside of therapy time:

‘I thought it was a good idea that they were getting extra practice, one of my initial concerns was the quality of the movement because we are always so concerned that we want to get them to move as biomechanically proper as possible…’ #PT2

‘…I would hesitate to even give it to someone at home. I mean, it’s meant to be a home program, but if all you’re doing is reinforcing that…that to use that tone to do it…I don’t think it’s benefiting them and so I would…I would hesitate to send someone home that’s not able to do it correctly.’ #OT10

### Cognitive participation & collective action

Therapists identified key individuals at each site who initiated and/or supported the implementation of GRASP. In six of the eight sites this individual was a clinical supervisor or practice leader:

‘I think the practice leaders did, so in OT practice we had two at the time, practice leaders who oversee all of OTs in the building and they really kind of initiated it…’ #OT4

‘…it helped to implement things pretty quickly because our professional practice leader, who oversees all the OT’s in a bunch of our sister Hospitals, she was very helpful and supportive…’ #OT13

As therapists had concerns about quality of unsupervised exercises, rehabilitation assistants were almost always involved in delivering GRASP to patients:

‘I don’t know if anyone has ever done them correctly the first time through, so I generally try to review it with them a couple of times if I can myself, I always do it once…I would get a rehab assistant to go over it with them a few times afterwards until they really could do them without sort of assistance.’ #OT5

‘… if they’re not doing the full booklet then she [the OT] will tell me which exercises she wants me to do with the patient and am from there if through working with them if I find that they’ve progressed or regressed I can, I’ll then let the OT know and we can either add or take away.’ #RA1

Acquiring the necessary equipment was identified as the most challenging process in implementing GRASP:

‘That was probably actually the biggest barrier and that’s probably why most therapists didn’t do it before because it is a lot of little tiny things that you need to collect…’ #OT5

‘We had one set of things to use as demonstration in our room in our gym but for the families they would have to go and individually buy all the stuff themselves.’ #PT2

In the four sites that were able to provide GRASP equipment, it was the rehabilitation assistants who were responsible for the process:

‘I actually stock pile all the equipment and we have little bags, like little back packs and we fill the right equipment for the right patient at that time…’ #RA2

‘So, they put together what are called starter kits at (one of the sites) and the RA’s, the rehab assistants, put them together. They chose items that were commonly used, but hard to get…’#OT1

### How the GRASP was used in practice

Therapists, when implementing GRASP in practice, have modified the intervention to fit with their clinical reasoning and the environment in which they work. A summary of how therapists’ use of GRASP differs from the intervention components identified within the GRASP Guideline Manual is shown in Table 2.

**Table 2:** **Therapists’ use of GRASP in clinical practice**

**Coverage (who should receive the intervention)**		
	Intervention component from GRASP Guideline Manual	Therapists use
1	Provide GRASP to stroke survivors in rehabilitation who can actively elevate their scapula against gravity and have palpatable wrist extension (grade 1); are aware of their safe bounds of ability; have sufficient cognition to be able to follow the programme; are able to report pain or fatigue	GRASP was reported to be used not only in stroke rehabilitation units but it is also used in acute care (n = 2), outpatient (n = 2), and community settings (n = 2); and with other population groups with neurological conditions.
		One therapist reported using the Fugl-Myer to select the appropriate GRASP level for each patient; the remainder selected the appropriate level based on observation of active movement and tone.
**Content (the content of the intervention)**		
	Intervention component from GRASP Guideline Manual	Therapists use
2	Provide a GRASP manual which includes unilateral and bilateral strengthening, range of motion, weight-bearing, and trunk control exercises along with gross and fine motor exercises	One therapist reported always providing the full GRASP manual to patients. The majority of therapists selected the most appropriate exercises from the manuals and printed them off individually.
3	Provide a variety of GRASP equipment which can be substituted	Two sites provide full kits of equipment, one site provides half sets of equipment which are the more difficult pieces to source (*e.g.*, donut weight for hand), one provides equipment piece by piece as needed, two use gym equipment that is cleaned and reused and two sell pieces of equipment to patients (*e.g.*, theraputty).
4	Provide a log sheet to monitor time spent completing exercises	Six therapists mentioned using/trying to use a written checklist or log sheet to monitor exercise completed. The remainder used verbal feedback from the stroke survivor and the clinical team to monitor whether or not exercises were being completed.
5	Progress to next GRASP level when the patient can complete over 50% of the exercises in the current level	As therapists do not always use the full GRASP manual, progression was discussed in terms of adding in new sheets of exercises or increasing repetitions as opposed to more structured progression through the levels of manuals.
6	Advise to complete the GRASP exercises outside of therapy time	Nine therapists reported that stroke survivors, where able, would be advised to complete exercises outside of therapy time. Barriers to prescribing exercises to be completed outside of therapy time included therapists’ beliefs about patients’ ability to correctly complete exercises, patient safety awareness, cognitive impairment and lack of family support for self-directed exercise. As a result GRASP exercises were most often completed with the supervision/assistance of a rehabilitation assistant.
7	Encourage to keep moving their paretic arm as best they can, improper movement should not be the cause of omitting an exercise	All therapists made references to concerns they had about the quality of the exercises that stroke survivors would do and the amount of compensation. Exercises are regularly modified or omitted if it was felt that they were not being done correctly—particularly exercises resulting in shoulder hiking.
8	Teach GRASP exercises to family/carers were possible	All therapists reported that family played an important role in GRASP. The readiness and willingness of family members, as determined by the therapists, would influence the extent to which they would be involved. A systematic approach to involving family members or carers in rehabilitation was not reported.
**Dose (frequency and duration)**		
	Intervention component from GRASP Guideline Manual	Therapists use
9	Advise to do the GRASP exercises for 60 minutes five times per week	Patients were advised by therapists to carry out the exercises as much as they could tolerate on a daily basis, rather than specifying 60 minutes daily. Therapists discussed different approaches to getting patients to complete the desired amount of practice, such as splitting GRASP up throughout the day and providing extra sessions with the rehabilitation assistant.

### Reflexive monitoring

Appraisal processes most often occurred at the level of the individual. Therapists were often only able to describe their own use and experiences with GRASP as opposed to more collective appraisal at a team or department level:

‘I’m not sure actually, I think a lot of people they just do it as it’s laid out, I’m not sure if there is the same level of customisation, am…but I can’t say for sure.’ #OT4

‘I think we all do our own thing…I don’t know if we all do it…I think we all sort of just tweaked it to what seems to work for us and our patients…’ #OT8

### Factors influencing the implementation and use of GRASP

The Consolidated Framework for Implementation Research (CFIR) was used to identify the most influential factors for using, and implementing, GRASP in practice. These factors are summarised in Table 3.

**Table 3:** **Factors influencing the implementation and use of GRASP**

Inner and outer setting	
Access to knowledge and information	Ten therapists reported that the GRASP website and free online availability of the treatment protocol enabled them to find out about the intervention and also facilitated its continued use.
Cosmopolitanism	Therapists reported finding out about GRASP through existing networks with the research team at GF Strong (where GRASP was developed) and national meetings with 11 therapists mentioning Janice Eng by name.
Leadership engagement	The implementation of GRASP was facilitated by active engagement of practice leaders and clinical supervisors as they were responsible both for identifying the programme and introducing it at the work site by acquiring resources to support implementation *e.g.*, funding for equipment.
Intervention characteristics	
Design, quality and packaging	GRASP was perceived to be well designed and presented. The large text and clear pictures were seen to be highly beneficial, particularly for a population often suffering from some degree of cognitive impairment. Therapists reported that the manual could be improved by shortening it and reducing repetition of exercises within and between levels of manuals.
Evidence strength and quality	All therapists agreed that GRASP was underpinned by best evidence for motor recovery after stroke and reported sharing this information with the patients to whom they prescribed GRASP.
Relative advantage	The primary advantage of GRASP was that it provided a more time efficient way of providing exercises to patients – something that therapists regularly do in practice anyway.
Complexity	Organising the GRASP equipment was identified as the most complex component of the intervention and this influenced the way in which the intervention was used *i.e.*, substituting items of equipment or omitting some exercises altogether.
Characteristics of individuals	
Knowledge and beliefs	Therapists’ beliefs about the quality of exercises that patients would be able to complete outside of therapy time influenced the way in which GRASP was used in practice *e.g.*, completing GRASP exercises during therapy time.

## Discussion

The aim of this study was to conduct a formative evaluation of the implementation of the Graded Repetitive Arm Supplementary Program to inform the development of a structured upper limb exercise program in the UK. The free online availability of the treatment protocol, along with well-established networks between the research and clinical teams, enabled therapists to find out about GRASP. All therapists expressed having positive first impressions of GRASP, but also reported that they had some concerns about prescribing exercises to be completed outside of therapy time. At each site, key individuals were identified who were responsible for driving the implementation of GRASP, and in the majority of sites this individual was the practice leader or clinical supervisor. All components of the GRASP intervention were modified to some extent when implemented in practice. Coverage was wider, the content adapted and the dose, when monitored, was less. Therapists, although providing comprehensive appraisal of the implementation and use of GRASP from their own perspective, were often unable to detail how GRASP was being used at a team or departmental level. Factors that emerged as influential for the implementation and use of GRASP have been identified.

The free online availability of the GRASP materials emerged as an important factor for therapists in finding out about the intervention. McCluskey *et al.* have identified that the paucity of detailed information on how to implement effective interventions acts as a barrier to implementing stroke guideline recommendations [21]. Within this article, it is suggested that researchers be required to make the protocols of effective interventions readily available to practitioners to overcome this barrier. Therapists most often found out about GRASP through existing internal and external networks with colleagues and the research team at GF Strong. Interestingly, the most frequent method of finding out about GRASP in the UK was also through colleagues [10]. Use of diverse formal and informal routes to acquire research knowledge has been previously reported [22] and is reflective of the ‘mindlines’ concept, in which healthcare professionals’ decision making is most often informed by ‘by their interactions with each other and with opinion leaders’ [23].

Therapists in this study identified key individuals at each site who took responsibility for driving the implementation of GRASP. In the majority of cases, these individuals were more senior therapists and practice leaders. A recent realist review on this topic investigated the complex interactions between change agents, knowledge utilisation, and work settings [24]. It was concluded that although evidence for the effectiveness of change agents was found to be weak, there was evidence to support the importance of these roles. However, a lack of systematic reporting of change agency interventions limited the conclusions that could be drawn, particularly in relation to personal characteristics of change agents and the extent to which they can be modified. More recently, Farley *et al.* have highlighted the challenge of collecting sufficiently detailed data to reliably and objectively identify high-quality opinion leaders within the health services [25]. It was our experience when travelling to the individual sites to conduct the interviews in this study that although the individuals responsible for introducing GRASP at each site were extremely enthusiastic about its implementation and use, it was evident from informal conversations that not all therapists at the site were as enthused by the program. This would lead us to believe that although the uptake and implementation of GRASP appears high in the province, the number of therapists within individual departments actually consistently using the intervention may not be as high, but to confirm this objectively a different study design is needed.

It is of particular interest to note in this study, that although the implementation of GRASP was found to be generally good, *i.e.*, all sites interviewed had successfully introduced GRASP to some extent into routine clinical practice, fidelity to the components outlined in GRASP Guideline Manual was lower than expected. It was found that all components of GRASP, when implemented in practice, were adapted to some extent to fit with therapists’ concerns about self-directed exercise and their working context. The multi-faceted nature of GRASP, and the design of the RCT in which it was tested, has meant that it has not yet been possible to determine which component(s), *i.e.*, those listed in Table 2, were the ‘active ingredient(s)’ and contributed to the overall success of the programme [7]. Harn *et al.* [26] have recently discussed this topic with respect to educational research and outline that interventions designed as a package that have been empirically tested become evidence-based practice when it is still unknown which components of the package are critical for success. Different schools of thought exist on adapting evidence-based interventions, but it is now known that adaptability of an intervention improves uptake and implementation [12,26,27].

In this study, therapists’ beliefs about self-directed practice emerged as one of the most influential factors for adapting GRASP when used in clinical practice. Despite the fact that the GRASP trial evidence showed that patients improved their function and movement quality [7], over one-half of the therapists expressed concerns that exercise completed without therapist supervision might result in poor-quality movement. These concerns stem in part due to long-standing, but unfounded, beliefs that practice of abnormal movement patterns promote poor movement quality [28]. This finding has parallels with fidelity studies from educational research where teachers’ individual teaching philosophy and concerns about interventions were found to moderate fidelity *i.e.*, teachers with more concerns about the value of the intervention demonstrated lower levels of implementation fidelity [29,30]. Divergent views of how an intervention fits with the role of those responsible for its implementation have also been identified as a significant barrier to implementation in two recent process evaluation studies using versions of NPT [31,32]. It is becoming increasingly evident that the congruence or ‘fit’ of an intervention with the beliefs of those delivering the intervention will determine success. Although non-adopters were not interviewed in this study, one could hypothesise that this perceived lack of congruence could, in part, explain non-adoption of GRASP in other sites. Is there a case to be made for addressing provider beliefs/concerns about evidence based interventions, or as Harn *et al.* suggest, is it time that empirically tested evidence-based interventions are adapted to better match individual contexts to optimise implementation fidelity [26]?

In recent years there has been a substantial increase in research studies seeking to influence professional practice. A number of Cochrane reviews exist that have aimed to establish the value of interventions such as computer generated reminders [33], printed educational materials [34], audit and feedback [35], and continuing education meetings and workshops [36]. An important finding in this study, when attempting to identify strategies to influence practice, was the level at which therapists appraised the implementation and use of GRASP. It was found that therapists were often unable to identify who in the department was using GRASP, and the way in which they were using the programme. Arguably it is difficult to influence service delivery, and therefore improve implementation fidelity, when service providers are not aware of what current practice is and do not benchmark or measure performance. This finding would suggest that there may be a role for self-monitoring, in the form of audit and feedback for example, to establish current practice and thus prompt fidelity to treatment guidelines. Audit and feedback has been shown to result in small but potentially important improvements [35] and recommendations as to how future empirical studies can further our understanding of the mechanisms of action of this complex intervention have been proposed [37].

### Use of implementation frameworks

As no one implementation framework was identified that could address all of the study objectives, three separate implementation frameworks were used to explore the processes of implementation, how GRASP is used in practice and emerging factors which influenced implementation and use. Using three implementation frameworks, although complicated, provided a systematic way of capturing the complex aspects of implementation.

NPT was useful in developing an interview guide to explore the implementation process at each site. However, using NPT alone did not allow for clear identification of the factors affecting these implementation processes or how the intervention was delivered, which are important for the purposes of a formative evaluation. Clarke *et al.* reported a similar finding when reflecting on their experience of using NPT in a process evaluation of the Training Caregivers after Stroke (TRACS) Trial [38]. NPT was said to place undue emphasis on individual and collective agency without acknowledging contextual factors that impacted on this agency. An unexpected finding when using NPT was therapists’ difficulty in trying to recall the processes involved in implementing GRASP at their work site. Indeed, even the practice leaders who often initiated the implementation of GRASP struggled to recall when they had first heard about the intervention. Therapists could often remember only the processes in which they as individuals were directly involved *e.g.*, a therapist would presume that it was the rehabilitation assistant that restocked the GRASP equipment box, but would report that they were not entirely certain. This fits with educational theories on learning in the workplace, which have found that ‘in everyday practices, learning takes place in the flow of experience, with or without our awareness of it’ [39].

Interviewing therapists in this study, as opposed to using audit or survey methodology, allowed us to get deeper insight into implementation fidelity and the reasons for adaptations to the intervention as opposed to just the way in which was adapted. The CFIF provided a comprehensive structure for reporting the use of the GRASP that will enable greater comparison across settings in the future. Domains from the CFIR were used to explain the research findings. The CFIR has been used previously using a ‘menu of constructs approach’ [40], where the focus has been on those factors relevant to the context of interest. When evaluating the implementation of a weight management program, using a cross-case comparison of ratings, ten CFIR constructs strongly differentiated the low versus high implementation facilities. The factors identified had parallels with our study, where networks and communications, leadership engagement, and relative advantage of the intervention were all found to influence implementation. This highlights the value of using such a framework, as consistent definitions allow for comparisons and synthesis of findings across studies.

Overlap between constructs was an issue, but the frameworks were not seen as mutually exclusive. Difficulties with commonality have been discussed by others, and it has been argued that irrespective of their coding, the use of frameworks helps to highlight important issues [17]. The flexible use of NPT has been applauded, as it demonstrates critical use of the constructs, rather than a ‘conceptual straitjacket’ [17,41]. We found the frameworks to be useful in ensuring comprehensiveness, but used them as complementary rather than restrictive guides to explore different aspects of the complex implementation elements. However, we did find the three frameworks were designed to be used from the perspective of the service provider without explicit consideration of the service-users. This is of particular importance for exploring implementation fidelity as characteristics of the recipients of interventions can often provide valid reasons for adaptations to interventions [29].

There is currently no gold standard or agreement as to which frameworks should be used, with a plethora available (*e.g.*, Flottorp *et al.* identified 12 frameworks and 57 factors that explored determinants of practice alone [42]). With existing frameworks being continually developed and refined, and the rapidly changing and advancing landscape of implementation science, there is as yet no clear guidance as to which framework to use and how it should be applied. Therefore we feel our approach was thorough and will inform the future intervention development.

### Limitations

The self-report data collected in this study relied on therapists’ ability to recall events from a few months to a couple of years prior to the interviews. Therefore one must be cautious when considering both the accuracy and the detail of these accounts. As participants in this study were volunteers, a self-selection bias exists where perhaps therapists with stronger opinions on the programme and/or its implementation are over represented thus limiting the generalisability of the study findings. The self-report nature of the data also introduces the risk of a social desirability bias where participants may have felt obliged to answer questions in a way that would be deemed pleasing to the researchers conducting the interviews. However, the research team both prior to, and during the interviews highlighted to participants that the data collected would be anonymised, and that it would not be possible for them to be identified in the hope that they would be as candid as possible.

### Practice implications and future research

To facilitate translation of effective interventions into routine clinical practice, it is of value to identify existing networks through which detailed information on how to implement the intervention can be communicated. Free online access to this information, in the case of GRASP, has been found to be highly effective. Implementation fidelity is moderated by providers’ beliefs or concerns about interventions. Co-creation of interventions during development, ensuring they are evidence-based but also best-fit to the providers’ beliefs and context, may help with implementation and fidelity. In addition to the intervention content, a behaviour change element and implementation strategy that facilitates the change in practice warrants further research. There is an urgent need for researchers to empirically test the ‘active ingredients’ of package interventions so that the mechanisms of action can be communicated to those responsible for their implementation. It is known that adaptability of interventions facilitates implementation. Therefore creative solutions that allow adaptation of intervention components while still delivering the active ingredients of interventions are required. As non-adopters of GRASP were not included in this study, we can only hypothesise possible reasons as to why this evidence-based intervention has yet to be implemented in more stroke rehabilitation units. Future research that objectively assesses actual uptake of interventions and explores factors influencing non-adoption of evidence-based interventions would provide further valuable information as to how interventions can be designed and adapted to improve congruence with therapists across settings.

## Conclusion

This study is particularly novel as data collection was not nested within a larger scale study to evaluate the intervention effectiveness. Instead, therapists and organisations in this region independently chose to adopt and implement this intervention, and have continued to use it long after the original research study was completed. This opportunity to explore what one could describe as ‘natural’ implementation of an intervention has provided a unique insight into how interventions translate from research trials into routine clinical practice. Although GRASP has translated into clinical practice, it is not used in the way in which it was shown to be effective. Novel therapist behaviour change interventions that are underpinned by theory may improve the implementation and fidelity of interventions to facilitate evidence-based practice. This formative evaluation has informed the development of a novel intervention that aims to bridge this evidence-practice gap in upper limb rehabilitation after stroke.

## Additional files

Additional file 1:
**Interview guide.** Interview guide developed for data collection (word document). Additional file 2:
**Coding frame.** Coding frame demonstrating how the three frameworks were used during data analysis (word document). Additional file 3:
**Recruitment of participants.** Breakdown of recruitment of participants across sites contacted (word document).
