# Supplementary material for: A formative evaluation of the implementation of an upper limb stroke rehabilitation intervention in clinical practice: a qualitative interview study
Source: Implement Sci. 2014 Aug 12;9:90. doi: 10.1186/s13012-014-0090-3 (PMC4156624; doi:10.1186/s13012-014-0090-3)
Supplement: Additional file 1: — Interview guide. Interview guide developed for data collection (word document). [file 13012_2014_90_MOESM1_ESM.docx]

**Additional file 1 – Interview guide**

**INTRODUCTORY QUESTIONS**

- Can you describe your role in stroke rehabilitation?
  - What is your current work setting?
  - What type of patients do you work with?

**FINDING OUT ABOUT GRASP**

- Can you tell us how you discovered the GRASP manuals?
- For how long have you been using GRASP in practice?

**USING GRASP**

- Can you describe in your own words how you use GRASP in practice?

*Prompts:*

- *Professions involved:*
  - What professions are involved in using GRASP at your work settings?
  - Have you had any training in using GRASP?
- *Choosing patients:*
  - Who do you use GRASP with?
  - How do you decide which manual to prescribe?
- *Providing the manual and equipment:*
  - Can you tell us about how you provide GRASP to patients?
  - At your work setting do you print off the whole manual or parts of the manual?
  - How do you decide how many repetitions to prescribe?
  - How do you go about communicating the prescribed exercises?
  - Are there any ways that you have changed or adapted the program?
  - How has your work setting gone about organising the GRASP equipment?
  - Do you think your colleagues use GRASP in the same way that you do?
- *Measuring adherence and outcomes:*
  - Do you have a way of monitoring how much exercise is being done by the patient?
  - Do you have a way of measuring if GRASP is effective?
- *Other:*
  - Is there anybody else you involve in using GRASP e.g. nursing staff, family?
  - Have you encountered any problems using GRASP?
  - Do you make any recommendations to patients when they are discharged from your service?

**PROCESSES OF IMPLEMENTING GRASP**

**Coherence**

- Is GRASP different from what therapists treating upper limb do on a daily basis anyway?
  - Does using GRASP make sense to the people you work with?
  - Do you think the purpose of GRASP is clearly conveyed in the manuals and in the online information provided?
  - Do the people you work with have a clear picture of the role they play in putting GRASP into practice?
  - Do the people you work with like GRASP?
  - Do they think implementing GRASP is worthwhile?

**Cognitive participation**

- Were there key people driving the implementation of GRASP?
  - Was there enough direction in getting going at the start?
  - Has there been sufficient involvement other professions in implementing GRASP?
  - Are some people more on board than others?
  - Did anything get in the way of implementing GRASP?
  - Does GRASP fit into your role?
  - Has using GRASP affected how your work is organised?

**Collective action**

- Are people able to carry out GRASP in practice?
  - Are people confident that GRASP is being implemented as it should be?
  - Have there been any changes to how GRASP is being implemented?
  - Do people have the right skills needed to implement GRASP?
  - Has there been any training provided?
  - Is there sufficient support from your work setting?
  - Is there anything in particular that supported the implementation of the programme?

**Reflexive monitoring**

- Do you think GRASP is working?
  - Do you have a way of measuring if GRASP is working or not?
  - Do the people you work with think it is worth continuing to use GRASP?
  - Will you continue to use GRASP in practice?
  - Would you/have you made any changes to GRASP based on your experiences?
