# Supplementary material for: A formative evaluation of the implementation of an upper limb stroke rehabilitation intervention in clinical practice: a qualitative interview study
Source: Implement Sci. 2014 Aug 12;9:90. doi: 10.1186/s13012-014-0090-3 (PMC4156624; doi:10.1186/s13012-014-0090-3)
Supplement: Additional file 2: — Coding frame. Coding frame demonstrating how the three frameworks were used during data analysis (word document). [file 13012_2014_90_MOESM2_ESM.docx]

## Additional file 2 – Coding frame

| **NORMALISATION PROCESS THEORY** | |
| --- | --- |
| **Coherence** | **Code** |
| References to differences or similarities in practice pre/post the introduction of GRASP  E.g. *We used to just do…, it’s different in the sense that now we…, Before we would never…* | Differentiation |
| References to differences in understanding between practitioners  E.g. *Not everybody agreed with…, some people felt that it should be…, it was difficult to establish consensus…* | Communal Specification |
| References to individual understanding of GRASP  E.g. *I felt it was very clear from the manual…, I’m not sure everybody understood their role…* | Individual specification |
| References to aspects of GRASP that are valued  E.g. *I really thought it was such a great idea to have…, it seemed like something families/carers would really value…* | Internalisation |
| **Cognitive Participation** | **Code** |
| References to influential people  E.g. *the senior therapist really advocated for the use of GRASP…, without the healthcare assistants it would have not have been feasible…* | Initiation |
| References to who should be involved (both staff and patients); suitability for involvement  E.g. *I really feel that implementing GRASP is the work of the …, I think we would benefit from input from…, There is certainly scope for further buy in from…* | Enrolment |
| References to conflicts in roles, people involved  E.g. *I think that the HCAs do not feel they should be responsible for supervising patients completing the GRASP exercises…, I think that there needs to be a more multi-disciplinary approach to delivering GRASP and that is lacking at present…* | Legitimation |
| References to methods of embedding the new practice in policies, procedures, processes  E.g. *We have dedicated time to ensure GRASP manuals/equipment are ready to be distributed…, We have changed our assessment proforma to include a section on GRASP…* | Activation |
| **Collective Action** | **Code** |
| References to the logistics of actually doing the work  E.g. *It was difficult not always having the equipment…, It is not always possible to have the family present when explaining GRASP…* | Interactional Workability |
| References to confidence in other people’s use of GRASP  E.g. *Sometimes I am concerned that other clinicians give the GRASP manuals to patients that are not suitable…* | Relational Integration |
| References to allocation of work to people  E.g. *I expected the physiotherapists to be more involved…, it would have been really helpful to have some training for all staff…* | Skill Set Workability |
| References to perceptions of organisational support  E.g. *We don’t have all the resources we need…, we are able to print everything we need and provide this to the patient to take home…* | Contextual Integration |
| **Reflexive Monitoring** | **Code** |
| References to how people are evaluating success  E.g. *All therapists use X outcome measure and we collate this data…, we don’t have a way of specifically evaluating GRASP…* | Systematization |
| References to the criteria used for evaluation  E.g. *As a department we can really see a change in ethos for upper limb exercises even in the acute setting…* | Communal Appraisal |
| References to reflection about whether the programme is worth doing for specific individuals  E.g. *I expected to be able to give GRASP to more patients, but that is not the case…* | Individual Appraisal |
| References to adaptations and changes that people  E.g. *Providing the equipment has been a challenge so we have removed some exercises from the manual…* | Reconfiguration |
|  | |
| **CONCEPTUAL FRAMEWORK FOR IMPLEMENTATION FIDELITY (CFIF)** | |
| References to what patients are provided GRASP  E.g. *I generally know after the first assessment if the patient is suitable…, I would never give GRASP to someone with shoulder pain…* | Coverage |
| References to how GRASP is provided  E.g. *I will try and have a family member present but that’s not always possible…, I often just give a handful of exercises instead of the whole manual…, I find patients generally don’t use the log sheet but I provide it always anyway…* | Content |
| References to the dose prescribed  E.g. *I will always ask patients to write down the numbers of exercises they do so I can progress the programme…, I advise patients to do a bit every day, as much as they can…* | Dose (Frequency/duration) |
|  | |
| **CONSOLIDATED FRAMEWORK FOR IMPLEMENTATION RESEARCH (CFIR)** | |
| **Intervention Characteristics** | **Code** |
| References to UBC, Janice Eng, Jocelyn Harris | Intervention Source |
| References to the evidence base for motor recovery after stroke, in particular increased intensity of repetitive task practice | Evidence Strength and Quality |
| References to the advantage of using GRASP  E.g. *I have found using GRASP saves so much time when it comes to prescribing exercises…, I can see that when the families have a structured programme they do a lot more with the patient* | Relative Advantage |
| References to how GRASP can be/has been modified | Adaptability |
| References to piloting of GRASP in the past | Trialability |
| References to difficulties implementing GRASP  E.g. *There was a lot of equipment to organise…, It wasn’t always possible to print off the full manual…* | Complexity |
| References to GRASP materials | Design Quality and Packaging |
| References to cost of using GRASP | Cost |
| **Outer Setting** | **Code** |
| References to patients needs as driver for change  E.g. *We are aware that increased attention to the upper limb is a priority for patients…,* | Patient Needs and Resources |
| References to networks with external organisations | Cosmopolitanism |
| References to perceived peer pressure or motivation  E.g. *All the sites in our area were using GRASP…,* | Peer Pressure |
| References to external incentives  E.g. *In the update of the clinical guidelines providing supplementary upper limb exercise has been highlighted…,* | External Policies and Incentives |
| **Inner Setting** | **Code** |
| References to layout of site, number of departments, level of administrative support, staffing etc. | Structural Characteristics |
| References to networks within the organisation  E.g. *The physio and OT departments work really well together here…* | Networks and Communications |
| References to norms, values and basic assumptions  E.g. *It is very usual for therapists to prescribe unsupervised exercise…* | Culture |
| ***Implementation Climate*** | ***Code*** |
| References to the fit of the intervention with therapists work  E.g. *We pride ourselves on being evidence-based and GRASP has been shown to be effective…* | *Compatibility* |
| References to the communication of goals  E.g. *The practice leaders were really clear what they wanted to achieve when introducing GRASP…* | *Goals & Feedback* |
| References to the climate for change  E.g. *I know we will be well supported even if we don’t see improvements straight away…* | *Learning Climate* |
| References to incentives within the organisation  E.g. *I was invited to deliver an in-service to demonstrate the work I had done on implementing GRASP…* | *Organizational Incentives & Rewards* |
| References to the priority placed on GRASP  E.g. *The focus in acute care will always be on transfers and mobilising…* | *Relative Priority* |
| References to the urgency for change  E.g. *We recognise the need to find ways for patients to do more therapy in stroke rehabilitation…* | *Tension for Change* |
| ***Readiness for Implementation*** | ***Code*** |
| References to ease of access to knowledge about GRASP  E.g. *The GRASP website has been a great way to access the manuals…* | *Access to Knowledge & Information* |
| References to dedicated resources to implementing GRASP  E.g. *We acquired some additional funding to put the equipment kits together…* | *Available Resources* |
| References to commitment and involvement of leaders  E.g. *It was the practice leader that first introduced GRASP and really advocated for its use…* | *Leadership Engagement* |
| **Characteristics of Individuals** | **Code** |
| References to individual attitudes towards an intervention  E.g. *I really felt that this additional exercise could make the world of difference to patients…* | Knowledge and Beliefs |
| References to individuals’ beliefs about their ability to implement GRASP  E.g. *I just wasn’t always confident that I was selecting the right patient for the GRASP…* | Self-Efficacy |
| References to stage of change an individual is in  E.g. *I had heard about GRASP and thought it was a good idea for a long time so was eager to use the programme…* | Individual Stage of Change |
| References to how individuals perceive the organisation  E.g. *I really feel like people are already under so much pressure here that it is not a good time to try and implement a new intervention…* | Individual Identification |
| References to other personal traits  E.g. *One of our colleagues is has considered returning to university to do an MSc and has a particular interest in research findings…* | Other Personal Attributes |
