# Supplementary material for: A formative evaluation of the implementation of an upper limb stroke rehabilitation intervention in clinical practice: a qualitative interview study
Source: Implement Sci. 2014 Aug 12;9:90. doi: 10.1186/s13012-014-0090-3 (PMC4156624; doi:10.1186/s13012-014-0090-3)
Supplement: Additional file 3: — Recruitment of participants. Breakdown of recruitment of participants across sites contacted (word document). [file 13012_2014_90_MOESM3_ESM.docx]

**Additional file 3 – Recruitment of participants**

| Site | Potential participants contacted: | Number invited | Number replied | Number interviewed |
| --- | --- | --- | --- | --- |
| A | Five therapists known to members of research team and thought to fulfil inclusion criteria | 5 | 5 | 5 |
| B | One therapist known to members of research team and thought to fulfil inclusion criteria | 1 | 1 | 1 |
| C | Two therapists who provided permission to be contacted about GRASP when downloading GRASP materials from website | 2 | 2 | 2 |
| D | One therapist who provided permission to be contacted about GRASP when downloading GRASP materials from website | 1 | 1 | 1 |
| E | Practice leader known to members of the research team, email forwarded to 8 other members of staff thought to fulfil inclusion criteria | 8 | 5 | 5 |
| F | Practice leader known to members of the research team, email forwarded to 3 other members of staff thought to fulfil inclusion criteria | 3 | 3 | 3 |
| G | Practice leader known to members of the research team, email forwarded to 3 other members of staff thought to fulfil inclusion criteria | 4 | 2 | 2 |
| H | Practice leader known to members of the research team, email forwarded to 10 other members of staff thought to fulfil inclusion criteria | 10 | 1 | 1 |
| I | Practice leader and two other members of rehabilitation team identified through BC registry contacted | 2 | 0 | 0 |
| J | Two therapists known to members of research team and thought to fulfil inclusion criteria | 2 | 2 | 0 |
| K | Two therapists identified through BC registry | 2 | 0 | 0 |
| L | One therapist provided permission to be contacted about GRASP when downloading GRASP materials from website. | 1 | 1 | 0* |
| Totals | | 42 | 23 | 20 |

*Replied after data collection had ceased but was willing to take part in interview
